# Predicting Ventral Striatal Activation During Reward Anticipation From Functional Connectivity at Rest

**DOI:** 10.3389/fnhum.2019.00289

**Published:** 2019-08-27

**Authors:** Asako Mori, Manfred Klöbl, Go Okada, Murray Bruce Reed, Masahiro Takamura, Paul Michenthaler, Koki Takagaki, Patricia Anna Handschuh, Satoshi Yokoyama, Matej Murgas, Naho Ichikawa, Gregor Gryglewski, Chiyo Shibasaki, Marie Spies, Atsuo Yoshino, Andreas Hahn, Yasumasa Okamoto, Rupert Lanzenberger, Shigeto Yamawaki, Siegfried Kasper

**Affiliations:** ^1^Department of Psychiatry and Neurosciences, Hiroshima University, Hiroshima, Japan; ^2^Department of Psychiatry and Psychotherapy, Medical University of Vienna, Vienna, Austria; ^3^Department of Neuropsychiatry, Graduate School of Medicine, The University of Tokyo, Tokyo, Japan; ^4^Health Service Center, Hiroshima University, Hiroshima, Japan

**Keywords:** ventral striatum, MRI, rest, monetary incentive delay task, prediction

## Abstract

Reward anticipation is essential for directing behavior toward positively valenced stimuli, creating motivational salience. Task-related activation of the ventral striatum (VS) has long been used as a target for understanding reward function. However, some subjects may not be able to perform the respective tasks because of their complexity or subjects’ physical or mental disabilities. Moreover, task implementations may differ, which results in limited comparability. Hence, developing a task-free method for evaluating neural gain circuits is essential. Research has shown that fluctuations in neuronal activity at rest denoted individual differences in the brain functional networks. Here, we proposed novel models to predict the activation of the VS during gain anticipation, using the functional magnetic resonance imaging data of 45 healthy subjects acquired during a monetary incentive delay task and under rest. In-sample validation and held-out data were used to estimate the generalizability of the models. It was possible to predict three measures of reward activation (sensitivity, average, maximum) from resting-state functional connectivity (Pearson’s *r* = 0.38–0.54 in validation data). Especially high contributions to the models were observed from the default mode network. These findings highlight the potential of using functional connectivity at rest as a task-free alternative for predicting activation in the VS, offering a possibility to estimate reward response in the broader sampling of subject populations.

## Introduction

Reward processing is an essential function of adaptive behavior in our everyday lives (Knutson et al., 2008). The ability to anticipate a positive incentive allows us to organize our behavior in accordance with the salience of the stimuli. Many researchers have investigated the neurobiological basis of reward processing using the well validated monetary incentive delay (MID) task in combination with functional magnetic resonance imaging (fMRI) across healthy and clinical populations (Lutz and Widmer, 2014; Oldham et al., 2018; Wilson et al., 2018).

The MID task allows for investigating the different stages of reward processing as well the different incentive amounts (Knutson et al., 2003). Specifically, activity in the ventral striatum (VS) during gain anticipation has been extensively investigated, particularly in relation to various aspects such as positive arousal (Wu et al., 2014) and reward sensitivity (van Hulst et al., 2015). The MID task has been used to uncover dysfunction in anticipation in various psychiatric disorders such as anhedonia in depression (Stoy et al., 2012; Takamura et al., 2017; Hoflich et al., 2018), schizophrenia (Grimm et al., 2014), attention-deficit/hyperactivity disorder (Plichta and Scheres, 2014), and in adolescents at risk of depression (Stringaris et al., 2015). The task has also been used in elderly people when investigating the aging-related alterations of motivation (Geddes et al., 2018). Thus, this task has the potential to be utilized as a trans-diagnostic indicator of neural reward anticipation across healthy population and psychiatric diseases. However, performance of the MID task may be quite difficult for some populations such as the elderly, children, and people with disabilities as these populations are not always capable of conducting tasks correctly. In addition, the implemented tasks may vary between institutions, thus causing comparability issues. Therefore, constructing a task-free method for evaluating neural reward processing would be crucial.

Resting-state fMRI (rsfMRI) has attracted attention as it does not require additional interfaces like a keyboard or screen compared to task-fMRI and allows investigating a broader sample participants almost irrespective of their physical and mental capabilities. Research has shown that rsfMRI captures each subject’s individual variability (Mueller et al., 2013) and could predict one’s brain activation during task-based fMRI (Tavor et al., 2016). However, as Tavor et al. (2016) used a gambling task the question remains unanswered whether VS activation to different reward amounts could be predicted using rsfMRI.

Here, we aimed to assess whether functional connectivity of the brain at rest allows for prediction of task-evoked VS activation during reward anticipation with the MID task. It might allow for predicting VS activation during reward anticipation without the need to acquire task-fMRI, potentially opening the doors to a useful approach to investigate neural reward function in various populations. We aimed to predict VS activation in regard to reward sensitivity, maximal reward, and average reward based on rsfMRI scans. Our focus was the prediction of reward sensitivity, as we previously reported that VS reward sensitivity could be a potential indicator for motivational anhedonia (Takamura et al., 2017). Reward sensitivity is the amount to which individual behavior is motivated by the relevant stimuli (Gray, 1987). Previously, an fMRI study using the MID task demonstrated proportional activation of the striatum in humans anticipating increasing financial gain in healthy individuals (Knutson et al., 2001). A meta-analysis also showed the proportional relationship between VS activity and subjective value of reward (Bartra et al., 2013). In this study, we consider VS reward sensitivity as the change in activation between increasing amounts of monetary gain. We also estimated this model for both, the maximal and average monetary reward, as previous studies mainly reported neural response to the highest or collapsed different values together (Oldham et al., 2018). Generalizability of our approach was further assessed using held-out validation data.

## Materials and Methods

### Participants

The subjects were recruited in three different locations as part of the healthy control cohorts in separate studies. For group 1 (Center of KANSEI Innovation), 17 participants were enrolled from the local community via a newspaper and public notice. Group 2 (Hiroshima University Hospital) included 15 participants who were similarly recruited as part of a previous study (Takamura et al., 2017). Group 3 (Kajikawa Hospital) contained 17 participants who were enrolled from freshmen attending Hiroshima University as part of a third study (Mori et al., 2016). All participants were right handed and examined by experienced psychiatrists and psychologists to exclude any history of brain disorders according to the DSM–IV criteria (American Psychiatric Association, 2000). To ensure psychiatric health, they were screened with the Mini International Neuropsychiatric Structural Interview (groups 1 and 2; Sheehan et al., 1998; Otsubo et al., 2005) or with the Japanese version of the Composite International Diagnostic Interview (group 3; Kessler and Ustun, 2004). None of the participants presented with a history of neurologic disorders, brain injury, and psychiatric disorders. Exclusion criteria comprised of a lifetime history of any psychiatric or neurological illness, taking psychiatric medications or undergoing psychotherapy, and any MRI contraindication. Of the 49 participants, three in group 1 and one in group 3 were excluded from the analysis due to artifacts in the resting state data [median percentage of removed spikes above 5%, according to Patel et al. (2014)]. Demographic characteristics of the 45 participants included in the analysis are as follows: Group 1 included 14 subjects (mean age ± standard deviation = 40.6 ± 5.7, seven males). Group 2 included 15 subjects (age = 44.9 ± 12.2, six males). Group 3 included 16 subjects (age = 19.1 ± 0.75, eight males). All the studies were carried out in accordance with the recommendations of Ethical Guidelines for Medical and Health Research Involving Human Subjects, the Ethics Committee of Hiroshima University with written informed consent from all participants. All subjects gave written informed consent in accordance with the Declaration of Helsinki and received financial reimbursement for participation. The protocols were approved by the Ethics Committee of Hiroshima University.

### Functional Image Acquisition

Functional brain images were collected using three different MRI scanners. All scans were acquired with echo planar imaging sequences. Details on MRI acquisition are summarized in Table 1. During rsfMRI, subjects were instructed to look at a central fixation point, lie still, stay awake, and not to think about anything specific.

**TABLE 1:** MRI acquisition parameters.

	**Group 1**		**Group 2**		**Group 3**	
**Site**	**Center of KANSEI Innovation**		**Hiroshima University Hospital**		**Kajikawa Hospital**	
**Scanner**	**3.0 T Siemens Magnetom Verio (Siemens, Erlangen, Germany)**		**3.0 T Signa HDxt Scanner (GE Healthcare, Milwaukee, WI, United States)**		**3.0 T Siemens Magnetom Spectra (Siemens, Erlangen, Germany)**	
**Type of scan**	**MID task**	**Resting-state**	**MID task**	**Resting-state**	**MID task**	**Resting-state**
FOV (mm)	240	212	192	256	192	192
Slice thickness (mm)	3.8	3.2	3	4	4	3
Slice gap (mm)	0.95	0.8	0	0	0	0
TR (ms)	2000	2500	2000	2000	2000	2700
TE (ms)	25	30	25	27	31	31
Slices	32	40	38	32	28	38
Flip angle (°)	80	80	90	90	90	90
Matrix size	64 × 64	64 × 64	64 × 64	64 × 64	64 × 64	64 × 64
Scan time (min:s)	2 × 12:10	10:00	12:20	05:00	2 × 12:10	05:00
Number of volumes (scans)	Two runs of 370	244	370	150	Two runs of 370	112

*MID task, monetary incentive delay task; FOV, field of view; TR, repetition time; TE, echo time.*

### MID Task

Participants performed a modified MID task as described by Knutson et al. (2003). The details of the task have been reported previously in our papers (Mori et al., 2016; Takamura et al., 2017). Before scanning, they were instructed how the task should be performed in a standardized manner and took part in one training session. Subjects were also informed that they would receive monetary reward equal to their performance in addition to the reimbursement for participation. For groups 1 and 3, the MID task consisted of two runs. Each run consisted of 90 trials with three conditions in a pseudo-randomized order (40 gain, 40 loss, and 10 neutral trials). In each trial, subjects could win or avoid losing 0, 20, 100, or 500 yen and were presented with one of nine types of cues indicating the trial condition (no response, 0 yen win/loss, 20 yen win/loss, 100 yen win/loss, or 500 yen gain/loss). This was followed by a fixation cross (anticipation phase), after which a target was briefly presented on the screen. If the subject pressed a button before the target offset, they gained or avoided losing the cued amount of money. Then a feedback showing the trial outcome was presented. Immediately after the feedback offset, the cue of the next trial was shown. The hit rate was adjusted to 66% for each subject by varying the allowed time to respond (Hahn et al., 2011). Due to limited scanner availability for group 2, the MID task consisted of one run, for a total of 90 trials with only two conditions in a pseudo-randomized order (62 win and 18 neutral trials). In the current analysis, only the gain-conditions were of interest. For groups 1 and 3, the two runs were concatenated to get approximately the same number of win trials as for group 2. After the fMRI session, participants were presented all types of cue stimuli and asked to evaluate their motivational levels for each stimulus conditions using a visual analog scale (VAS). The collected responses of VAS ratings were translated to 0–100%.

### Behavioral Data Analysis

As the behavioral data were skewed, we used non-parametric descriptive statistics. Friedman test was used to compare the effect of the reward amount (¥0, ¥20, ¥100, or ¥500) on subjective motivational levels and reaction times. The Dunn–Bonferroni correction was used as *post hoc* analysis. The behavioral data analysis was done using SPSS statistics 21 software (IBM, Armonk, NY, United States).

### fMRI Data Preprocessing

Preprocessing was conducted using Statistical Parameter Mapping (SPM8) software (Wellcome Department of Cognitive Neurology, Wellcome Trust Centre, London, United Kingdom). We discarded the first five volumes of each run for the MID task to ensure magnetic signal equilibrium. For each rsfMRI data in groups 2 and 3, the scans acquired during the first 14 s were discarded; the first seven volumes for group 2 (TR = 2000 ms) and five for group 3 (TR = 2700 ms; the TR for the MID task was the same for all groups). As the data in group 1 belonged to the Brain Mapping by Integrated Neurotechnologies for Disease Studies^1^, the scans acquired during the first 10 s were discarded; the first four volumes (TR = 2500 ms) according to the standardized pipeline. All imaging data were preprocessed using slice-timing correction and subsequently two-pass realignment to the mean image. Then, using the normalization parameters obtained through the segmentation of the structural image which was previously aligned with the mean functional image, the data were normalized to the standard template on the Montreal Neurological Institute (MNI) reference brain, and resampled to 2 × 2 × 2 mm^3^ voxels. The MID task data were then smoothed using an 8 mm full-width at half-maximum (FWHM) Gaussian kernel. In order to standardize the length of all rsfMRI scans between cohorts, the data from group 1 were shortened to the first 5 min for the subsequent analysis. We created a gray matter mask by combining and thresholding the respective compartments of Harvard-Oxford atlas and SPM tissue probability map at 50%, which was applied to the rsfMRI images. Afterward, artifacts were further removed using the Brain Wavelet toolbox^2^ (Patel et al., 2014) with the “threshold” parameter set to 15 due to the application on unsmoothed data.

### fMRI General Linear Model: MID Task

For groups 1 and 3, we modeled 24 regressors that included the 8 reward anticipation conditions [amount of money (± ¥0, 20,100, 500)], 2 control events (neutral cue and neutral feedback), and 14 outcome phase activities [six conditions (± ¥20,100,500) × two outcome (hit/miss) and two conditions (± ¥0)]. For group 2, the first-level included 14 regressors that modeled anticipating reward in the four reward cue conditions [amount of money (+ ¥0, 20,100, 500)], eight feedback phase activities [four conditions (+ ¥0, 20,100, 500)] × two outcome (hit/miss)], and two control events (neutral cue and neutral feedback). In detail, the following aspects of the experiment were modeled: For group 2, there was hit or miss feedback for the 0 yen condition. For groups 1 and 3, there was just one common feedback for both, hit and miss, outcomes of ±0 yen. We used these feedbacks to improve the stability of the model for groups 1 and 3, in which the trial numbers for one block were less than those of group 2. All condition regressors were convolved with canonical hemodynamic response function. The six realignment parameters for each subject were furthermore included as nuisance regressors. Further details are provided in our previous papers (Mori et al., 2016; Takamura et al., 2017).

Since we focused on gain anticipation, individual parameter estimates were generated for four contrasts: *small reward anticipation*: 20 yen anticipation vs. 0 yen anticipation, *medium reward anticipation*: 100 yen anticipation vs. 0 yen anticipation, *maximal reward anticipation*: 500 yen anticipation vs. 0 yen anticipation, and *average reward anticipation*: 20,100, and 500 yen vs. 0 yen anticipation.

For extraction of the VS activation, we used the peak coordinates of gain anticipation areas from a meta-analysis of the MID task (Oldham et al., 2018), which are *x*/*y*/*z* = 12/10/−4, −10/10/−6 in MNI space. We defined spheres around these maxima with a radius of 6 mm and merged the bilateral ventral striata into one ROI using MarsBaR (Brett et al., 2002). The overlapping region between the gray matter mask and these ROIs served as a mask for the subsequent extraction of contrast estimates. To identify the general activation patterns evoked by reward anticipation, we conducted one-sample *t*-tests for the contrasts of the maximal reward and average reward anticipation by applying a significance threshold of *p* < 0.05 with a peak family-wise false positive error (FWE) correction. For this analysis, the group mask was constructed by combining the gray matter mask (see the section “fMRI Data Preprocessing”) and all task-fMRI volumes of all subjects as an inclusive mask.

### Reward Sensitivity Parameter

Based on our previous study (Takamura et al., 2017) we parameterized neural reward sensitivity by modeling VS activity during gain anticipation as a function of the reward rank. These neural activations were fitted using a linear regression model.

Y=b+0bR1

***Y*** represents the VS activation, *R* the reward rank (1 = 20 yen, 2 = 100 yen, 3 = 500 yen), *b*_1_ the slope, and *b*_0_ the constant term. Of note, we used the rank instead of reward magnitude which was used in the previous paper, due to the more linear relationship between the rank and the VS activation. The slope indicates the sensitivity to benefit size, where a larger slope parameter (*b*_1_) indicates higher reward sensitivity.

### rsfMRI Data Processing

After the common preprocessing, further rsfMRI-specific processing comprised artifact reduction by motion, tissue, and frequency regression, and estimation of the FC. White matter (WM) and cerebrospinal fluid (CSF) masks were created using the respective compartments of the Harvard-Oxford atlas with a 95% threshold. Nuisance regressors were defined as the first three principal components of WM and CSF (Behzadi et al., 2007) and the six realignment parameters to control for movement artifacts in the resting-state data. Bandpass filtering from 0.01 to 0.1 Hz was conducted using sine and cosine regressors in the same model as recommended by Hallquist et al. (2013). We applied these additional data cleaning process because resting state data are more vulnerable to different sources of artifacts (Birn, 2012; Huijbers et al., 2017). Whole-brain functional connectivity was calculated as Pearson correlation between the mean time series extracted from spheres of 6 mm radius around the coordinates provided in the ROI set of Power et al. (2011). We used the ROIs set by Power et al. (2011) because it was created from both task and RS results. Hence, we expect there is an inherent relationship to task activation. No thresholding was necessary as the ROI set only specifies coordinates and no probability of voxels belonging to any parcel (as, e.g., in the Harvard-Oxford atlas). Negative connectivities were included in the model as they are since we consider them to be integral parts of the network organization [e.g., between the attention and the default mode (DM) network]. The ROIs for extraction of the mean signal were defined as 10-mm-spheres. To confirm the general functional connectivity patterns, an elementwise inference on the mean connectivity matrices over all 45 subjects was conducted. Taking the correlations between functional connections into account, the significances were estimated by the permutation test with 10,000 runs (null hypothesis: 0 connectivity value) to a two-sided FWE-corrected threshold of *p* ≤ 0.05.

### Relevance Vector Regression

We aimed to predict three different measures of VS activation: reward sensitivity, maximal reward, and average reward. In order to build a model, we used relevance vector regression (RVR) (Tipping, 2001) implemented in PRoNTo^3^ (Schrouff et al., 2013). In the current analysis, the data were mixed between the sites and randomly split into an estimation (*n* = 30) and held-out samples (*n* = 15) equally distributed over all cohorts in order to show generalizability of our results. The generalizability of the model was estimated using a 10-fold cross-validation (10-FCV), repeatedly splitting the estimation sample into training (*n* = 27) and test sets (*n* = 3) for model estimation. We trained one model for each measure of the VS activation to learn associations between the patterns of functional connectivity at rest and the targeted VS measure. The individual predictors were calculated using the weight and individual functional connectivity matrices for each subject. A regression model was employed to correct the scaling of the predicted and actual value, which are not automatically accounted for by PRoNTo. The weight matrix averaged over the folds and the complete estimation set was used for this. The individual predictors were fed into the resulting regression model. We report Pearson’s correlation coefficient and the mean squared error (MSE) as measures of similarity between the predicted and actual values of the VS measures. An MSE of 0 would indicate no error between the predicted and actual values. For the performance on the training runs, the significances were estimated by the permutation test implemented in PRoNTo with 10,000 runs. Resulting *p*-values < 0.05 were considered significant. We also present the nodes and edges with the highest weight vector values that contribute the most to estimations. The ROIs were grouped to seven functional networks according to Yeo et al. (2011) for simplification; visual (VI), somato-motor (SM), dorsal attention (DA), ventral attention (VA), fronto-temporal (FT), fronto-parietal (FP), DM network (DMN), and two anatomical regions; basal ganglia (BG) and cerebellum (Harvard Oxford atlas).

## Results

### Behavioral Results

There was a significant difference in subjective motivational levels on the amount of money [χ^2^(3) = 42.067, *p* < 0.01]. *Post hoc* tests revealed significant differences between all conditions (*p* < 0.05) after Bonferroni adjustment, indicating that participants were motivated more by increasing monetary rewards. There was a significant difference in reaction times on the amount of money [χ^2^(3) = 101.273, *p* < 0.01]. Dunn–Bonferroni *post hoc* tests were carried out and there were significant differences between 0 and 100 yen, 0 and 500 yen, or 20 and 500 yen (*p* < 0.01). The difference between 20 and 100 yen is marginally significant (*p* = 0.054). There was no significant difference between other amount of money (*p* > 0.1), which might be due to the flooring effect for 100 and 500 yen. The median response time and motivation ratings are shown in Table 2.

**TABLE 2:** Summary of the behavioral data.

	**Amount of money**			
	**0 yen**	**20 yen**	**100 yen**	**500 yen**
Subjective motivational levels (%), median (interquartile range)	48 (37)	63 (25)	76 (22)	99 (16)
Reaction time (ms), median (interquartile range)	244.9 (41.5)	235.5 (38.8)	228.6 (39.5)	228.2 (46.3)

*Due to skewed distributions, non-parametric measures have been used.*

### Neuroimaging Results

**FIGURE 1:**
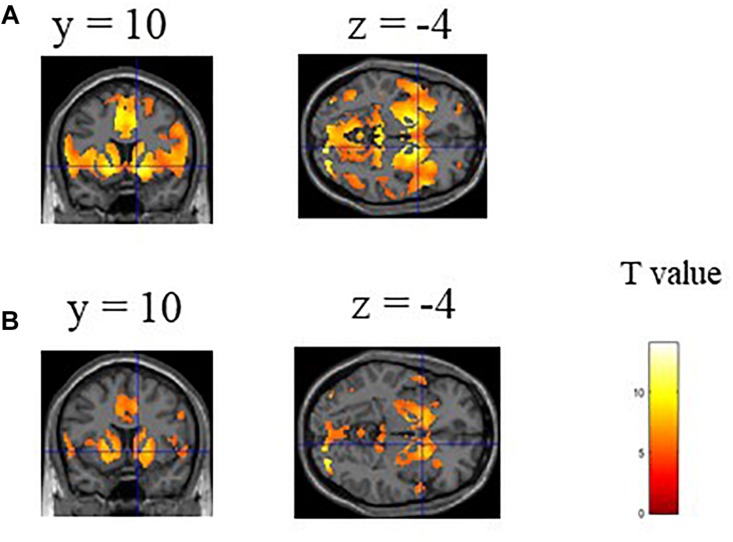
Statistical parametric maps of brain regions associated with **(A)** maximum and **(B)** average reward. One sample *t*-test of 45 subjects showed significant VS activation. A significant threshold was considered *p* < 0.05, with a peak family-wise false positive rate (FWE) corrected.

**FIGURE 2:**
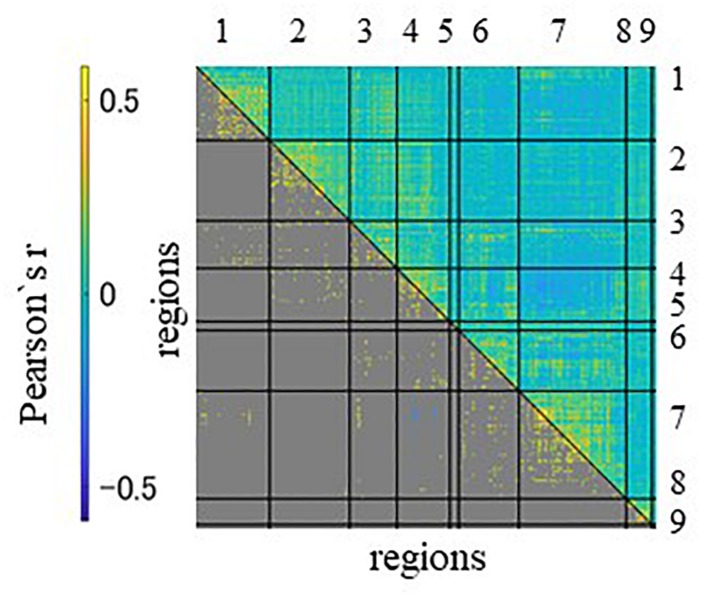
The region-with-region functional connectivity matrix of 45 subjects, sorted by network assignment. The upper triangle contains all connectivity, the lower only the significant ones. The significances were estimated by the permutation test with 10,000 runs. A significant threshold was considered *p* < 0.05, with a peak family-wise false positive rate (FWE) corrected. Columns and rows represent regions of interest (ROIs) for resting state, which are grouped according to seven networks and two anatomical regions. Networks and anatomical regions from left to right and top to bottom as follows: 1, visual; 2, somato-motor; 3, dorsal attention; 4, ventral attention; 5, fronto-temporal; 6, fronto-parietal; 7, default mode networks; 8, basal ganglia; and 9, cerebellum. Diagonal and non-diagonal sections show functional connectivity within- and between-network for these ROIs, respectively.

We first confirmed the brain activations related to anticipation of the maximum and average reward across all participants. As shown in Figure 1, a one-sample *t*-test indicated activation of large clusters including the VS, the prefrontal, and occipital and temporal cortices which confirmed the validity of the experiment. We next showed the average functional connectivity matrix across all subjects to inspect the general patterns of functional connectivity at rest. As shown in Figure 2, regions within the same networks correlated positively.

### Relevance Vector Regression

**FIGURE 3:**
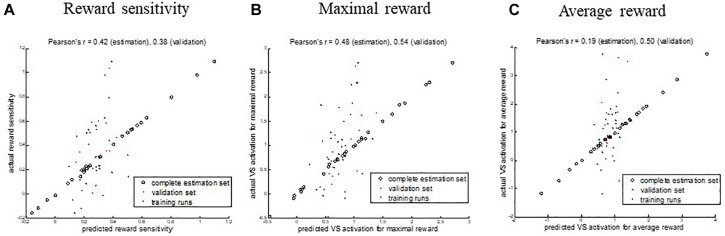
Correlations between the predicted and actual values of ventral striatum (VS) activation from functional connectivity at rest for three different measures. **(A)** Reward sensitivity, **(B)** maximal reward, and **(C)** average reward. Open circles denote the results of the complete estimation set. Black dots show the performance of the single training runs. Red dots show the performance on the validation sample. Scaling and offset errors were corrected using linear regression.

The model performance of the training runs and the validation with the held-out sample showed that the similarities between the predicted and actual values for the maximal and reward sensitivity measures were comparable: Reward sensitivity − Pearson’s *r* = 0.42 (*p* = 0.001), MSE = 0.08 (*p* = 0.003) for estimation, Pearson’s *r* = 0.38 (*p* = 0.16), MSE = 0.06 (*p* = 0.09) for validation, maximal reward − Pearson’s *r* = 0.48 (*p* = 0.003), MSE = 0.49 (*p* = 0.006) for estimation, Pearson’s *r* = 0.54 (*p* = 0.04), MSE = 0.38 (*p* = 0.02) for validation. For the average reward, the performance on the held-out sample was higher than for the training runs: Pearson’s *r* = 0.19 (*p* = 0.13), MSE = 1.02 (*p* = 0.21) for estimation, Pearson’s *r* = 0.50 (*p* = 0.06), MSE = 1.09 (*p* = 0.03) for validation (Table 3 and Figure 3). It needs to be emphasized that the *p*-value alone provides little information on the model performance due to the different sample sizes in both sets but the correlation coefficients can be interpreted as effect sizes, which are by definition independent of the sample size. Figure 3 shows the predicted and the original data for the three models. The predicted values of the complete estimation set forming almost a straight line only indicate that the training process was successful.

**TABLE 3:** Correlation coefficients and MSE between the actual and predicted VS measures.

	**Measures of VS activation**					
	**Reward sensitivity**		**Maximal reward**		**Average reward**	
	**Pearson’s *r* (*p*-value)**	**MSE (*p*-value)**	**Pearson’s *r* (*p*-value)**	**MSE (*p*-value)**	**Pearson’s *r* (*p*-value)**	**MSE (*p*-value)**
Performance of training runs (*n* = 30)	0.42 (0.001)	0.08 (0.003)	0.48 (0.003)	0.49 (0.006)	0.19 (0.13)	1.02 (0.21)
Performance on validation with held-out sample (*n* = 15)	0.38 (0.16)	0.06 (0.09)	0.54 (0.04)	0.36 (0.02)	0.50 (0.06)	1.09 (0.03)

*MSE, mean squared error; VS, ventral striatum.*

**FIGURE 4:**
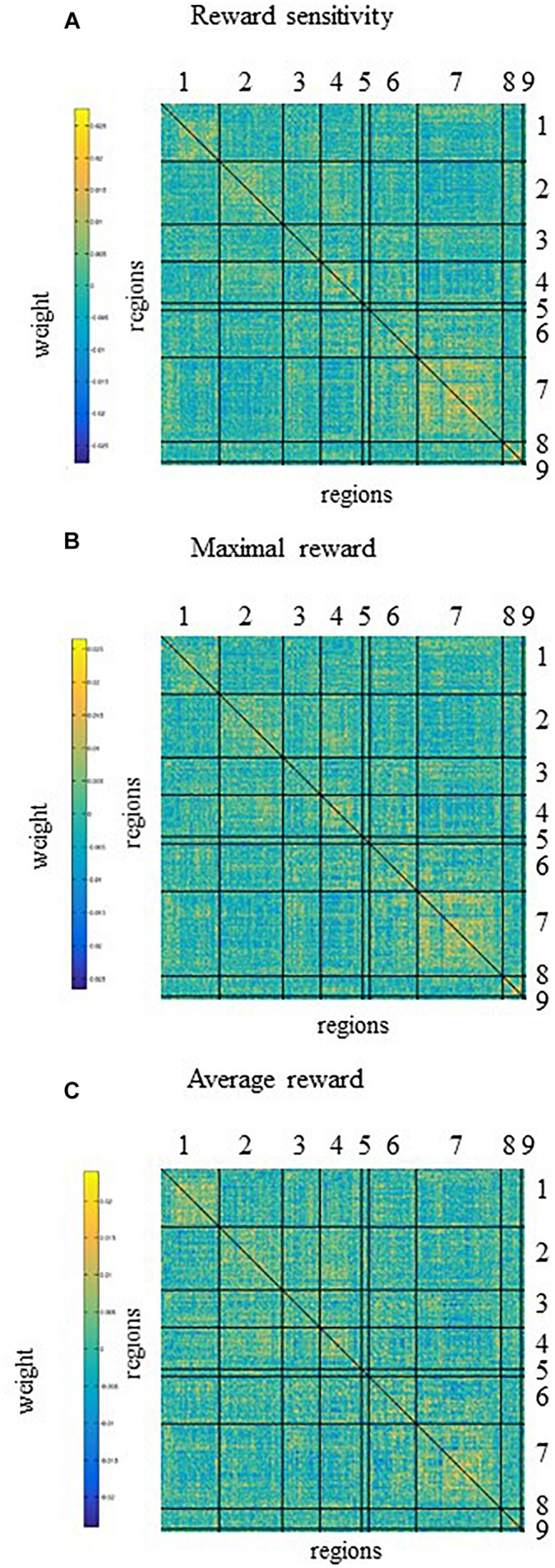
The averaged weight matrices of contributions of functional connectivity across the whole brain to prediction of **(A)** reward sensitivity, **(B)** maximal reward, and **(C)** average reward. Columns and rows represent regions of interest (ROIs) for resting state, which are grouped according to seven networks and two anatomical regions. Networks and anatomical regions from left to right and top to bottom as follows: 1, visual; 2, somato-motor; 3, dorsal attention; 4, ventral attention; 5, fronto-temporal; 6, fronto-parietal; 7, default mode networks; 8, basal ganglia; and 9, cerebellum. Diagonal and non-diagonal sections show functional connectivity within- and between-network for these ROIs, respectively. For all models **(A–C)**, most of the functional connectivity with high weight correspond to the functional connectivity within or between the default mode network and other networks. There is a high similarity between the averaged weight matrices for reward sensitivity **(A)** and maximal reward **(C)** in terms of the participation of different networks, less for the average reward model.

Five and four out of the top nodes are located in the DMN for reward sensitivity and maximal reward, respectively, where three of them are present in both models (Figure 4 and Tables 4, 5). The node with highest weight (*x*/*y*/*z* = −46/−61/21) is also located in the DMN and corresponds to the temporal parietal junction (TPJ). For the average reward model, two out of the top five nodes are located in the DMN (Figure 4 and Table 6). The nodes with the highest and second highest contribution are located in the VA network and correspond to the supplementary motor cortex and insular cortex, respectively (Table 6). Overall, most of the edges with strong influence for the three models are related to the DMN (Figure 4 and Tables 4–6). Figure 4 shows the averaged weight matrices for all three models. Columns and rows represent ROIs for resting state, which are grouped according to seven functional networks and two anatomical regions as detailed in the section “rsfMRI Data Processing.” Tables 4–6 list the top five nodes (highest weight sum) and edges (highest single weights) for the predictors. As shown in Figure 4 and Tables 4, 5, there is a high similarity between the averaged weight matrices for reward sensitivity and maximal reward model in terms of the participation of different networks (less for the average reward model). Indeed, reward sensitivity also correlated positively with the VS response to the maximal gain (Pearson’s *r* = 0.87, *p* < 0.01).

**TABLE 4:** Top five nodes and edges with highest weights for the predictors of reward sensitivity.

	**Nodes**				**Edges**							
**Rank**	**Coordinate of the node**			**Network**	**Coordinate of node 1**			**Network**	**Coordinate of the node 2**			**Network**
	***x***	***y***	***z***		***x***	***y***	***z***		***x***	***y***	***z***	
1	–46	–61	21	DM	43	–72	28	DA	–46	–61	21	DM
2	65	–12	–19	DM	–10	39	52	DM	–58	–26	–15	DM
3	–58	–26	–15	DM	–2	38	36	DM	–58	–26	–15	DM
4	–2	38	36	DM	–58	–30	–4	DM	–58	–26	–15	DM
5	–16	29	53	DM	–16	–77	34	DM	15	–87	37	VI

*VI, visual; DA, dorsal attention; VA, ventral attention; DM, default mode.*

**TABLE 5:** Top five nodes and edges with highest weights for the predictors of maximal reward.

	**Nodes**				**Edges**							
**Rank**	**Coordinate of the node**			**Network**	**Coordinate of node 1**			**Network**	**Coordinate of the node 2**			**Network**
	***x***	***y***	***z***		***x***	***y***	***z***		***x***	***y***	***z***	
1	–46	–61	21	DM	43	–72	28	DA	–46	–61	21	DM
2	65	–12	–19	DM	–2	38	36	DM	–58	–26	–15	DM
3	–2	38	36	DM	56	–46	11	DM	31	33	26	VA
4	43	–72	28	DA	–20	45	40	DM	–2	–37	44	DM
5	–2	–37	44	DM	59	–17	29	VA	–53	–10	24	SM

*SM, somato-motor; DA, dorsal attention; VA, ventral attention; DM, default mode.*

**TABLE 6:** Top five nodes and edges with highest weights for the predictors of average reward.

	**Nodes**				**Edges**							
**Rank**	**Coordinate of the node**			**Network**	**Coordinate of node 1**			**Network**	**Coordinate of node 2**			**Network**
	***x***	***Y***	***z***		***x***	***y***	***z***		***x***	***y***	***z***	
1	–3	2	53	VA	–20	64	19	DM	–3	42	16	DM
2	–34	3	4	VA	13	30	59	DM	6	64	22	DM
3	65	–12	–19	DM	–2	38	36	DM	–11	45	7	DM
4	8	–72	11	DA	24	45	–15	FP	–7	–71	42	DM
5	56	–46	11	DM	56	–46	11	DM	31	33	26	VA

*DA, dorsal attention; VA, ventral attention; FP, fronto-parietal; DM, default mode.*

## Discussion

The main goal of this study was to investigate whether functional connectivity at rest could predict task-evoked VS activation during reward anticipation. The results showed that a multivariate pattern of functional connectivity at rest can predict VS activation for maximal/average reward anticipation and reward sensitivity assessed via the MID task in a healthy population. Furthermore, the DMN was shown to play a critical role in this process. The models derived within the current work provide a method for estimating VS activation for the extensively validated MID task without the need to acquire task fMRI, which might provide a way to assess neural reward function in people not capable of performing the required tasks correctly.

For the maximal reward and reward sensitivity models, the predictive power of the performance on the validation sample is comparable to that of training runs [Pearson’s *r* = 0.54 (validation set), 0.48 (training runs) for maximal reward, Pearson’s *r* = 0.38 (validation set) and 0.42 (training runs) for reward sensitivity]. For the average reward model, the predictive power of the performance on the validation sample (Pearson’s *r* = 0.50) is much higher than that of training runs (Pearson’s *r* = 0.19) and comparable to that of the maximal reward model (Pearson’s *r* = 0.54). This might be caused by greater heterogeneity within the training than the validation sample and indicates potential predictive capabilities of the model beyond significance.

As for the reward sensitivity and maximal reward models, the node with the highest contribution to prediction of the VS measures was located in the DMN. In detail, the node corresponds to the TPJ, which is a heteromodal associative region with structural connections to the limbic system, the prefrontal cortex, the cingulate gyrus, the putamen, and the thalamus (Chafee and Goldman-Rakic, 2000; Kucyi et al., 2012). This enables the TPJ to work as a hub by integrating information from the external and internal environment and processing it when necessary. In relation to reward processing, a previous study showed that the TPJ encoded the value and salience of the stimuli (Kahnt et al., 2014). That the TPJ was assigned the highest weight for the maximum reward and the sensitivity model might thus be explained by its role as a network hub. For the average reward model, the nodes with the highest and second highest contribution are located in the VA network and correspond to the supplementary motor cortex and the insular cortex, respectively. A meta-analysis of the MID task revealed that the supplementary motor cortex and the insula were activated during anticipating averaged over all amounts of money, as well as maximal and minimum amount of money (Oldham et al., 2018). This indicates that those regions are recruited for anticipation in general, independent of the relative magnitude of potential reward.

For all models, most of the edges with the highest weights correspond to the functional connections within the DM, and between the DM and other task-positive networks such as the DA and the VA networks. This suggests that the DMN itself contributes highly to the prediction of VS measures from functional connectivity at rest. The weight matrix by itself does not tell whether these networks have increased or decreased functional connectivity in relation to the VS measures. Still, these findings may help us to understand a comprehensive role of the DMN to interact with other networks for predicting VS measures during anticipation.

Considering the high correspondence between resting state connectivity and task activation (Smith et al., 2009; Cole et al., 2014), the fact that the salience network (SN) does not really light up as predictor might be surprising. Still, the SN might indirectly have an influence through regulating the DMN, instead of directly predicting the VS activation, as the SN mediates switching between the DMN and the network regarding task execution (Sridharan et al., 2008).

The idea that brain connectivity at rest can predict activation during a task has previously been reported by using the Human Connectome Project dataset (Tavor et al., 2016). The authors showed that the predictive power of functional connectivity at rest for task-fMRI activation (correlation coefficient between predicted and real values) was around 0.7. Tavor et al. (2016) used a gambling task in which participants guessed whether the number written on a card was higher than 5 or not and they were blinded to the amount of money they could earn or lose in each trial (Delgado et al., 2000). The gambling task had only one gain anticipation condition. Hence, there were no equivalents of sensitivity and maximal reward. This is to some extent equal to the average reward in the current analysis. Thus, the task used in Tavor et al. (2016) does not allow for the investigation of VS activation in proportion to reward amount.

The proportional response in the VS to increasing amount of gain (reward sensitivity) is disrupted in depression (Takamura et al., 2017). If generalizable across various psychiatric disorders and healthy population, VS reward sensitivity has a potential to be a trans-diagnostic indicator of gain anticipation.

A previous study tried to predict neural activation in the striatum with a reinforcement learning task through the use of structural connectivity (Smittenaar et al., 2017). The authors divided the striatum into the four regions, namely bilateral caudate and putamen and examined the striatal response during motor response, the expected value at outcome phase and the reward response. In the respective task, participants were required to respond to a slot machine by pressing a button. Finally, the outcome was presented indicating a reward or not. Their predictive power (correlation coefficient between predicted and real value) reached 0.20, where the target region was the right putamen and the contrast was the expected value during the outcome phase. In comparison, using functional connectivity as a predictor, our results showed that the correlation coefficients between estimated and original data were 0.38–0.54 in the validation sample. One possible reason for this may be that functional connectivity at rest might be closer to brain activation during tasks than structural connectivity as predictor since they are based on the same type of signal.

A major merit of the current study is the possibility of in-sample as well as out-of-sample validation. Although it is common to use cross-validation only, ideally a trained model should be tested with independent samples. In addition, given that participants were scanned at multiple sites with different scanners, we showed that the model could learn a predictive pattern across datasets.

Our results need to be interpreted in the light of methodological limitations. The length of the resting state scans is comparably short and the repetition times are relatively long. Even though the overall analyzed resting state period is within the appropriate range according to a recent study (from 5 up to 13 min) (Birn et al., 2013), longer scan durations and a shorter TR could improve the quality of the model by adding stability to the functional connectivity estimates. In addition, though the rationale of the current study was to show that predicting measures of ventral striatal activation from functional connectivity at rest is possible, a larger sample size would be helpful for further model optimization (e.g., inclusion of different imaging modalities). Lastly, the current study might also have implications for clinical research. If the models derived within the current study can be generalized beyond healthy subjects, this approach might form a way to evaluate reward response in people not able to perform the MID task.

## Data Availability

The data that support the findings of this study are available from the corresponding author upon reasonable request.

## Ethics Statement

All the studies were carried out in accordance with the recommendations of Ethical Guidelines for Medical and Health Research Involving Human Subjects, the Ethics Committee of Hiroshima University with written informed consent from all subjects. All subjects gave written informed consent in accordance with the Declaration of Helsinki and received financial reimbursement for participation. The protocols were approved by the Ethics Committee of Hiroshima University.

## Author Contributions

AM, MK, GO, RL, YO, SYa, and SK contributed to the conception and design of the overall analysis which was coordinated by AM and MK. MK, AM, and MT performed the statistical analysis planned by RL, MK, AM, MR, and AH. AM, GO, KT, SYo, CS, and AY performed the recruitment. AM, GO, MT, KT, SYo, NI, CS, and AY conducted the initial medical screening. AM, GO, MT, KT, SYo, NI, CS, and AY recorded the neuroimaging data. AM, GO, MT, and CS performed the quality control of the data. AM, CS, and AY provided the medical assistance and subject monitoring. GO, YO, and SYa provided the medical supervision. AM, MK, MR, MS, GO, MT, GG, AH, RL, SK, SYo, MM, YO, PH, and PM interpreted the data. AM composed the overall manuscript. MK provided parts of the sections “Materials and Methods” and “Results.” All authors have critically revised the manuscript, and read and approved the submitted version of the manuscript.

## Conflict of Interest Statement

SK received grants and research support, consulting fees, and/or honoraria within the last 3 years from Angelini, AOP Orphan Pharmaceuticals AG, Celgene GmbH, Eli Lilly, Janssen-Cilag Pharma GmbH, KRKA-Pharma, Lundbeck A/S, Mundipharma, Neuraxpharm, Pfizer, Sanofi, Schwabe, Servier, Shire, Sumitomo Dainippon Pharma Co. Ltd., and Takeda. RL received conference speaker honorarium within the last 3 years from Shire and support from the Siemens Healthcare regarding clinical research using PET/MR. MS has received travel grants from Janssen, Eli Lilly, Austroplant, and AOP Orphan Pharmaceuticals AG; speaker honoraria from Janssen and Austroplant; and workshop participation from Eli Lilly. She is also the recipient of a NARSAD Young Investigator Grant from the Brain & Behavior Research Foundation (23741). YO has received lecture fees from Otsuka, Dainippon-Sumitomo, Astellas, Pfizer, Eli Lilly, Janssen, Meiji, Mochida, Yoshitomi, Eisai, and GSK. The remaining authors declare that the research was conducted in the absence of any commercial or financial relationships that could be construed as a potential conflict of interest.

## Footnotes

1 http://brainminds-cro.umin.jp/translable_brain_marker_protocols.html

2 http://www.brainwavelet.org/

3 http://www.mlnl.cs.ucl.ac.uk/pronto/
